# Characteristics associated with fruit and vegetable consumption in mid aged and older Chinese men and women: a cross-sectional analysis of the first wave of WHO SAGE China

**DOI:** 10.1017/S000711452400151X

**Published:** 2024-09-28

**Authors:** Justine Derbyshire, Sarah A. McNaughton, Karen E. Lamb, Catherine Milte

**Affiliations:** 1 School of Exercise and Nutrition Sciences, Deakin University, Geelong, VIC, Australia; 2 Institute for Physical Activity and Nutrition (IPAN), School of Exercise and Nutrition Sciences, Deakin University, Geelong, VIC, Australia; 3 Health and Well-being Centre for Research Innovation, School of Human Movement and Nutrition Sciences, University of Queensland, St Lucia, QLD, 4067, Australia; 4 Melbourne School of Population and Global Health, University of Melbourne, Parkville, VIC 3052, Australia

**Keywords:** Fruit and vegetable consumption, Older adult, China, Socio-ecologic determinants

## Abstract

Adequate fruit and vegetable consumption is essential for healthy ageing and prevention and management of chronic disease. This study aimed to examine characteristics associated with fruit and vegetable consumption in Chinese men and women aged 50 years and over. Data from the first wave of the Chinese cohort (2008–2010) of the WHO’s Study on global AGEing and adult health (SAGE) survey was used. Fruit and vegetable consumption was assessed by self-reported typical consumption in serves/day. Characteristics examined were age, education, financial security, home ownership, marital status, social cohesion and rural location. Associations with fruit and vegetable consumption were assessed using multiple linear regression adjusted for confounders and stratified by sex. Overall, women consumed more serves of fruit per day than men (mean (standard deviation): 2·6 (2·2) and 2·2 (2·1) serves/day, respectively) whereas men consumed more serves of vegetables than women (7·2 (4·0) and 6·7 (3·7)). Lower fruit consumption was associated with lower education, lower social participation, income insecurity, renting, being un-partnered and rural residency in men and women, as well as older age in women. Lower vegetable consumption was associated with older age, lower education and urban residency in men and women and lower social participation in men and being unpartnered in women. This study has identified characteristics associated with fruit and vegetable intake in a sample of mid aged and older Chinese men and women. Further research on the interrelationships between these characteristics and fruit and vegetable intake as well as longitudinal relationships is warranted.

China is expected to have the highest growth rate of older adults worldwide in the next 12 years^(1)^. Approximately 337·5 million Chinese residents (25·3 %) were estimated to be over the age of 50 years in 2010 and given the projected growth rate of 71 %, this population is predicted to increase to approximately 573·8 million by the year 2050^(1,2)^. Increasing age is associated with a greater risk of developing lifestyle-related chronic diseases resulting in increased healthcare costs^(3,4)^. Adequate fruit and vegetable consumption is essential for healthy ageing and in the prevention and management of chronic, age-related disease^(5,6)^. The Chinese Dietary Guidelines recommend adults consume 150–300 g of fruit and 300–500 g of vegetables per day for optimal health^(7)^. In 2009, approximately 34 % of older Chinese adults met vegetable recommendations whereas only 5 % met fruit recommendations^(8)^. In recent years, China has experienced increased urbanisation and commercialism, which has seen a transition from a traditional diet including rice, vegetables and meats to a more Westernised diet composed of higher intakes of meat, dairy, fruits and highly processed foods^(9)^.

Identifying the determinants of fruit and vegetable consumption is an important step in developing public health programmes and interventions to increase consumption^(10,11)^. The socio-ecological model of health behaviours is a theoretical model that considers the multifaceted determinants of health behaviours across intrapersonal, interpersonal, community and societal domains^(12)^.

While determinants of fruit and vegetable intake in older adults have been widely studied in high-income countries (HIC), more research in low- and middle-income countries (countries with a Gross National Income <$US12235 per capita per year) is needed. Few studies have investigated determinants of fruit and vegetable consumption in adults aged over 50 years in middle-income countries^(13–32)^, with little research conducted in China^(30)^. Previous studies in both high- and middle-income countries have reported that being female, being married, having a higher level of education and higher income may be associated with higher fruit and vegetable intake in older adults^(13–19,21,22,24–32)^. However, Li *et al.* reported that Chinese men were more likely to meet fruit and vegetable recommendations compared with women^(30)^. Socio-economic factors are important determinants of health that have also been extensively investigated as potential determinants of fruit and vegetable intake in HIC. Given that variations in economic transition and inequalities contribute to variations in determinants of health, further investigation is warranted^(33)^. While social support and participation has been shown to be an important determinant of fruit and vegetable intake in HIC and points to a sex-specific relationship between the two variables, it has not been examined to the same extent in low- and middle-income countries^(18,19,22,23,25,27–32)^. Existing research suggests that area of residency, that is urban *v*. rural location, may be an important influence on health and health behaviours and may be particularly important to examine in contexts undergoing economic transition and urbanisation^(10,19,30,32)^.

Given the changing demographics and rate of ageing in China and the limited research available examining the Chinese population, the aim of this study was to investigate characteristics associated with fruit and vegetable consumption among Chinese men and women aged 50 years and over.

## Methods

The WHO’s Study on global AGEing and adult health (SAGE) is an ongoing longitudinal study investigating the health of adults, primarily those aged 50 years and over with a smaller comparison cohort of adults aged 18–49 years, in six low- and middle-income countries. SAGE was approved by the WHO’s Ethical Research Committee and Shanghai Municipal Center for Disease Control^(34)^. The first wave of data for the Chinese cohort was collected from 2008 to 2010 and is available open access on request for research. Of the 15 050 adults aged 18 years and over approached for the survey, 14 813 agreed to participate, the majority were aged 50 years and over (*n* 13 175)^(34)^. The current study consists of secondary analysis of data collected in the 50 years and over cohort. Recruitment was conducted using a three-stage random sampling procedure which has been described elsewhere^(34)^. Households were invited to participate from one of eight provinces and sixteen strata selected to provide coverage of geographical location, urban and rural settings and socioeconomic level. All participants from a household classified as a ‘50+ year household’ were invited to participate. A single household questionnaire was completed for each household followed by an individual face to face interview for each person aged 50 years and over. Participants provided individual informed consent to participate prior to the interview. For individuals who could not complete the interview due to potential memory problems, a proxy was identified to complete the interview on their behalf. The survey was conducted in Chinese^(34)^.

### Fruit and vegetable consumption

Participants were asked ‘How many servings of (fruit/vegetables) do you eat on a typical day?’ and were shown a flash card indicating a typical serve (e.g. medium piece of fruit, half a cup of fruit/vegetable juice, half a cup of raw or cooked vegetables including tomato, potato and beans, 1 cup leafy greens). Responses for fruit and vegetables were recorded separately and analysed separately^(32,35)^.

### Individual and environmental characteristics

Characteristics potentially associated with fruit and vegetable consumption were examined across the individual, social environment and physical environment levels of the socio-ecological framework^(12)^.

### Individual level

Participant age in years was recorded by the interviewer^(35)^. Age was considered as both a continuous variable and in categories (50–59 years, 60–69 years, 70–79 years and 80+ years,) to assess potential non-linearities. Education was assessed by asking if respondents had ever received formal schooling then (if yes) to state the highest level of education they had received. Education was classified into six categories: ‘No formal schooling’, ‘Less than primary’, ‘Completed primary’, ‘Completed secondary’, ‘Completed high school’ and ‘Completed college/university/post-graduate studies’^(35)^. Perceived financial security was assessed by asking if respondents felt they had enough money to afford all necessities and obligations. Reponses were classified as ‘No’ or ‘Yes’^(35)^. Home ownership was assessed by asking respondents ‘Is the dwelling you live…’ possible answers included owned and paid in full by oneself or a member of the household, owned and still being paid off by oneself or a member of the household, rented or other. Responses were categorised as ‘Family-owned’ including owned and paid, and owned and being paid by either the respondent or a household member, ‘Rented’ or ‘Other’ (including living with friends or living in government-provided homes)^(36)^.

### Social environment level

Respondents were asked to state their marital status. Responses for the SAGE survey included married, co-habiting, divorced, widowed, separated or never married. For this study, responses were categorised as ‘Un-partnered’ (defined as being separated, divorced, widowed or never married) and ‘Partnered’ (defined as being currently married or co-habiting)^(35)^.

Social participation was assessed by developing a social cohesion index based on nine questions in SAGE. Respondents were asked to identify how often they attended public meetings; met community leaders; attended any club, group, society, union or organisational meeting; worked with people in the neighbourhood; had friends over to their home; been in the home of someone who lives in a different neighbourhood than you do or had them in your home; socialised with co-workers and attended religious services or attended social meetings, activities, programs events or visited friends or relatives outside the home. Responses to each of these questions ranged from 1 ‘Never’, 2 ‘Once or twice per year’, 3 ‘Once or twice per month’, 4 ‘Once or twice per week’ and 5 ‘Daily’^(35)^. The responses to these questions were summed to obtain an index of social cohesion ranging from 9 to 45, where a higher social cohesion score was associated with higher levels of social participation.

### Physical environment level

Location was categorised as either ‘Urban’ or ‘Rural’ and recorded by the interviewer^(35)^.

### Other covariates

Language was determined by asking respondents ‘What is your mother tongue?’ as a part of the socio-demographic section of the individual questionnaire^(35)^. Responses were categorised into ‘Chinese, Mandarin’, ‘Chinese, Other’ and ‘Other’.

Chronic disease status was assessed by asking participants if they had been diagnosed with stroke, angina and diabetes and additional questions relating to history of suffering from temporary paralysis or loss of feeling and ongoing chest pains upon exertion in the past 12 months. The additional questions were used as a proxy for diagnosis of stroke and angina, respectively, in the absence of an official diagnosis^(35)^. Self-reported health status was assessed using the question ‘In general, how would you rate your health today?’ Responses were categorised as ‘Good’, ‘Moderate’ or ‘Bad’^(35)^.

An adapted twelve-question WHO Disability Assessment Schedule and fourteen additional questions regarding difficulty with activities of daily living or instrumental activities of daily living were used to assess disability. Participants whom reported severe or extreme difficulty in one or more items from the WHO Disability Assessment Schedule, activities of daily living or instrumental activities of daily living were defined as having severe/extreme disability^(35)^.

BMI was calculated from height and weight measures taken by the interviewer^(35)^. Participants were categorised into one of four categories: ‘Underweight’ (<18·5 kg/m^2^), ‘Normal Weight’ (18·5–22·9 kg/m^2^), ‘Increased Risk’ (23·0–27·5 kg/m^2^) or ‘High Risk’ (>27·5 kg/m^2^), according to WHO guidelines for BMI in south-east Asian populations^(37,38)^. BMI was also analysed as a continuous variable in the present study.

Alcohol consumption was assessed using the questions ‘Have you ever consumed a drink that contains alcohol?’ and ‘Have you consumed alcohol in the past 30 days?’. Alcohol consumption was divided into three categories: ‘No’ (never), ‘Yes, Not Recently’ (has consumed alcohol, not in the past 30 days) and ‘Yes’ (has consumed alcohol in the past 30 days)^(35)^. Smoking status was assessed using two questions: ‘Have you ever smoked tobacco or used smokeless tobacco?’ and ‘Do you currently use any tobacco products?’. Responses were categorised as ‘Yes’ or ‘No’^(35)^.

The Global Physical Activity Questionnaire was used to assess physical activity^(35,39)^. Respondents were asked fifteen questions relating to physical activity at work, during leisure time and active transport. Responses were recorded and converted to minutes per day. For this study, moderate-vigorous physical activity was calculated by adding the total time spent in the following five domains: moderate and vigorous activity at work, moderate and vigorous activity during leisure time and active transport (walking and cycling). Sedentary behaviour was determined by asking respondents ‘How much time do you usually spend sitting or reclining on a typical day?’. Responses were recorded and converted into minutes per day^(35)^.

### Statistical analysis

All statistical analysis was conducted using Stata 15·1^(40)^. Only participants with complete data on fruit and vegetable consumption and each of the potential individual and environmental characteristics of interest were included in this study. Participant data were excluded if they were younger than 50 years or had an incomplete dataset. Participant characteristics were summarised using mean and standard deviation (s d) for continuous and frequency (*n*) and percentage (%) for categorical variables.

Associations between each of the individual and environmental characteristics and fruit or vegetable consumption were assessed using multiple linear regression models. For each characteristic, two regression models were considered: a crude model and a model adjusting for potential confounders. Directed acyclic graphs were developed to assist in identifying potential confounders of each exposure and outcome relationship for the seven potential correlates considered (see online Supplementary Information Fig. 1–7 illustrating directed acyclic graphs for the fruit intake outcome; the vegetable intake directed acyclic graphs were the same). Wald tests were undertaken to examine the overall significance of categorical characteristics examined. As associations may differ for men and women, all models were stratified by sex. SAGE survey weightings were applied in the models.

## Results

Of the 13 175 WHO SAGE participants aged 50 years and over, 9541 (72 %) had complete data for analysis (4450 men and 5091 women) (Table 1). Men had a slightly higher average social participation score than women (15·0 (s d = 3·6) *v*. 14·8 (s d = 3·5), respectively). More men resided in rural areas (53·2 % *v*. 46·5 %). Overall, men reported higher levels of alcohol consumption (54·0 % *v*. 11·5 %), smoking (54·2 % *v*. 3·6 %) and physical activity (764 (s d = 766) *v*. 654 (s d = 670) minutes per day) compared with women. In contrast, women were more likely to have no formal education (33·8 % *v*. 13·2 %), be unpartnered (21·6 % *v*. 10·5 %), suffer from angina (16·4 % *v*. 12·5 %) and be severely or extremely disabled (20·6 % *v*. 15·9 %). Women also reported a higher mean BMI (24·0 (3·6) kg/m^2^ *v*. 23·4 (3·2) kg/m^2^) and were more likely to be classified as ‘High Risk’ (BMI > 27·5 kg/m^2^) (16·8 % *v*. 10·4 %). No differences were found for age, language, financial security, stroke, diabetes or sedentary behaviour between men and women.

**Table 1.** Descriptive characteristics of older adults in China, SAGE wave 12 008–2010. (*n* 9541)

	Overall (*n* 9541)		Men (*n* 4450)		Women (*n* 5091)	
	Mean or *n*	sd or %	Mean or *n*	sd or %	Mean or *n*	sd or %
Age, y, mean (sd), range	62·9, 50–95	9·3	63·0, 50–95	9·2	62·8, 50–93	9·2
Age, y, n (%)						
50–59	4225	44·3	1940	43·6	2283	44·8
60–69	2847	29·8	1341	30·1	1505	29·6
70–79	1983	20·8	941	21·2	1042	20·5
80+	469	4·9	228	5·1	261	5·1
Fruit consumption, serves/day, mean (sd)	2·4	2·2	2·2	2·1	2·6	2·2
Vegetable consumption, serves/day, mean (sd)	6·9	3·9	7·2	4·0	6·7	3·7
Education, n (%)						
No formal education	2305	24·2	586	13·2	1719	33·8
Less than primary	1733	18·2	820	18·4	913	17·9
Completed primary	1883	19·7	1046	23·5	837	16·4
Completed secondary	1998	20·9	1071	24·1	927	18·2
Completed high school	1223	12·8	665	14·9	558	11·0
Completed college/uni/post-grad	399	4·2	262	5·9	137	2·7
Financially secure, n (%)						
Yes	6757	70·8	3150	70·8	3607	70·9
Home ownership^a^, n (%)						
Family-owned	8439	88·5	3977	89·4	4462	87·7
Rented	583	6·1	261	5·9	322	6·3
Other	519	5·4	212	4·7	307	6·0
Marital status, n (%)						
Partnered	7973	83·6	3982	89·5	3991	78·4
Social participation^b^, mean (sd)	14·9	3·6	15·0	3·6	14·8	3·5
Location, n (%)						
Urban	4808	50·4	2083	46·8	2725	53·5
Language, n (%)						
Chinese, Mandarin	9508	99·7	4438	99·7	5070	99·6
Chinese, other	27	0·3	8	0·2	19	0·4
Other	6	0·1	4	0·1	2	0·0
Angina, n (%)						
Yes	1390	14·6	554	12·5	836	16·4
Diabetes, n (%)						
Yes	623	6·5	238	5·4	353	6·9
Stroke, n (%)						
Yes	468	4·9	238	5·4	230	4·5
Disability status, n (%)						
Severe/Extreme	1755	18·4	706	15·9	1049	20·6
Self-rated health, n (%)						
Good	3210	33·6	1643	36·9	1567	30·8
Moderate	4396	46·1	2001	45·0	2395	47·0
Bad	1935	20·3	806	18·1	1129	22·2
BMI, kg/m^2^, mean (sd)	23·7	3·4	23·4	3·2	24·0	3·6
BMI^c^, kg/m^2^, n (%)						
Underweight	423	4·4	199	4·5	224	4·4
Normal	3729	39·1	1892	42·5	1837	36·1
Increased risk	4070	42·7	1897	42·6	2173	42·7
High risk	1319	13·8	462	10·4	857	16·8
Alcohol consumption, n (%)						
No	6552	86·7	2047	46·0	4505	88·5
Yes, not recently	987	10·3	714	16·0	273	5·4
Yes	2002	21·0	1689	38·0	313	6·1
Smoking status, n (%)						
Yes	2599	27·2	2414	54·2	185	3·6
Physical activity (moderate-vigorous), min/week, mean (sd)	706	718	764	766	654	670
Sedentary behaviour, min/week, mean (sd)	1558	939	1547	963	1568	917

a Home ownership ‘other’ refers to households where the home is not owned by family (or the respondent) and rent is not paid, for example in government-provided accommodation, living with friends or living with an unrelated carer.

b The social participation score was calculated based on responses from nine categories (community meetings, meeting with leaders, visiting friends and relatives, going out, attending religious services, attending groups/clubs, visiting others and socialising with co-workers and neighbours). Possible range from 9 to 45, with higher participation scores reflecting greater social participation.

c BMI was classified as: Underweight (<18.5 kg/m^2^), normal (18.5–22.9 kg/m^2^), increased risk (23.0–27.5 kg/m^2^) and high risk (>27.5 kg/m^2^)^(37,38)^.

### Fruit and vegetable consumption

The mean (s d) consumption of fruit and vegetables for all participants was 2·4 (2·2) and 6·9 (3·9) serves/day, respectively. On average, women had a higher fruit consumption than men (2·6 (2·2) serves/day and 2·2 (2·1) serves/day, respectively). However, men reported a higher mean vegetable consumption than women (7·2 (4·0) serves/day and 6·7 (3·7) serves/day, respectively).

### Associations between individual and environmental characteristics and fruit and vegetable consumption

#### Fruit consumption

Increasing age was associated with lower fruit consumption in women (*β* = –0·03, 95 % CI) = –0·04, –0·02) (Table 2). However, this association was not found in men. Compared with women aged 50–59 years, women aged 60–69 years, 70–79 years and 80+ years had lower fruit consumption (Table 2). After adjusting for age and rural residency, men and women with higher levels of education were both found to have higher average fruit consumption (e.g. completed college/university/post-grad *v*. no formal education: *β* = 1·33, 95 % CI = 0·86, 1·81 and *β* = 2·31, 95 % CI = 1·58, 3·04, respectively). Wald tests identified significant overall associations between categorical age and fruit consumption for women (F(1,48) = 45·29, *P* < 0·001) and education and fruit consumption in both men and women (F(1,48) = 53·50, *P* < 0·001 and F(1,48) = 48·62, *P* < 0·001, respectively). In both men and women, being financially secure after adjusting for age, education and marital status (*β* = 0·50, 95 % CI = 0·31, 0·70 and *β* = 0·53, 95 % CI = 0·32, 0·75), being partnered after adjusting for age (*β* = 0·73, 95 % CI = 0·45, 1·00 and *β* = 0·31, 95 % CI = 0·07, 0·55) and having higher social cohesion scores after adjusting for age, marital status and rural residency (*β* = 0·07, 95 % CI = 0·04, 0·11 and *β* = 0·10, 95 % CI = 0·08, 0·13) were associated with higher average fruit consumption. Living in a rental property after adjusting for age, education, marital status and rural residency and rural residency after adjusting for age was associated with lower average fruit intake in men (*β* = –0·33, 95 % CI = –0·62, –0·05 and *β* = –1·10, 95 % CI = –1·34, –0·86, respectively) and women (*β* = –0·74, 95 % CI = –1·07, –0·40 and *β* = –1·44, 95 % CI = –1·66, –1·22, respectively).

**Table 2.** Findings from linear regression models of associations between socio-ecologic characteristics and fruit consumption (serves/day) by sex in older adults from WHO SAGE China, 2007–2010 (weighted) (*n* 9541)

	Men (*n* 4450)		Women (*n* 5091)	
	Crude	Adjusted	Crude	Adjusted
	*β* (95 % CI)	*β* (95 % CI)	*β* (95 % CI)	*β* (95 % CI)
Age (continuous)	–0·00(–0·01, 0·01), *P* = 0·824	n/a	–0·03(–0·04, –0·02), *P* < 0·001***	n/a
Age		n/a		n/a
50–59 years	ref		ref	
60–69 years	–0·11 (–0·28, 0·06), *P* = 0·195		–0·24 (–0·43, –0·05), *P* = 0·015*	
70–79 years	0·07 (–0·21, 0·36), *P* = 0·610		–0·50 (–0·80, –0·20), *P* = 0·001**	
80+ years	0·02 (–0·47, 0·52), *P* = 0·933		–1·24 (–1·53, –0·94), *P* < 0·001***	
Education^a^				
No formal education	ref	ref	ref	ref
Less than primary	0·26(–0·01, 0·53), *P = 0·*062	0·25 (–0·01, 0·51), *P* = 0·055	0·33 (0·11, 0·54), *P* = 0·003**	0·17 (–0·04, 0·39), *P* = 0·111
Completed primary	0·74 (0·50, 0·98), *P* < 0·001***	0·58 (0·35, 0·82), *P* < 0·001***	0·95(0·75, 1·14), *P* < 0·001***	0·55(0·36, 0·75), *P* < 0·001***
Completed secondary	0·78 (0·51, 1·05), *P* < 0·001***	0·51(0·24, 0·77), *P* < 0·001***	1·35 (1·12, 1·58), *P* < 0·001***	0·78 (0·53, 1·03), *P* < 0·001***
Completed high school	1·55 (1·15, 1·94), *P* < 0·001***	1·15(0·79, 1·51), *P* < 0·001***	2·08(1·70, 2·47), *P* < 0·001***	1·36 (0·92, 1·80), *P* < 0·001***
Completed college/university/post-grad	1·96 (1·49, 2·44), *P* < 0·001***	1·33 (0·86, 1·81), *P* < 0·001***	3·08 (2·41, 3·74), *P* < 0·001***	2·31 (1·58, 3·04), *P* < 0·001***
Financial security^b^				
No	ref	ref	ref	ref
Yes	0·70 (0·46, 0·94), *P* < 0·001***	0·50 (0·31, 0·70), *P* < 0·001***	0·84 (0·57, 1·11), *P* < 0·001***	0·53 (0·32, 0·75), *P* < 0·001***
Home ownership^c^,^d^				
Family owned	ref	ref	ref	ref
Rented	0·17 (–0·12, 0·46), *P* = 0·257	–0·33(–0·62, –0·05), *P* = 0·024*	–0·19(–0·51, 0·14), *P* = 0·256	–0·74 (–1·07, –0·40), *P* < 0·001***
Other	0·70(0·26, 1·15), *P* = 0·003**	0·34 (–0·07, 0·75), *P* = 0·098	0·61 (0·30, 0·92), *P* < 0·001***	0·29 (–0·10, 0·68), *P* = 0·136
Marital status^e^				
Unpartnered	ref	ref	ref	ref
Partnered	0·71 (0·44, 0·98), *P* < 0·001***	0·73 (0·45, 1·00), *P* < 0·001***	0·55 (0·35, 0·75), *P* < 0·001***	0·31 (0·07, 0·55), *P* = 0·012*
Social participation^f^,^g^	0·05(0·01, 0·09), *P* = 0·009**	0·07(0·04, 0·11), *P* < 0·001***	0·09(0·06, 0·12), *P* < 0·001***	0·10(0·08, 0·13), *P* < 0·001***
Location^h^				
Urban	ref	ref	ref	ref
Rural	–1·08 (–1·33, –0·84), *P* < 0·001***	–1·10 (–1·34, –0·86), *P* < 0·001***	–1·39 (–1·60, –1·17), *P* < 0·001***	–1·44 (–1·66, –1·22), *P* < 0·001***

* *P* value < 0.05.

** *P* < 0.01.

*** *P* < 0.001.

a Education model adjusted for age and urban/rural residency.

b Financial security model adjusted for age, education, marital status and urban/rural residency.

c Home Ownership ‘Other’ refers to households where the home is not owned by family (or the respondent) and rent is not paid, for example in government-provided accommodation, living with friends or living with an unrelated carer.

d Home ownership model adjusted for age, education, financial security, marital status and urban/rural residency.

e Marital status model adjusted for age.

f The social participation score was calculated based on responses from nine categories (community meetings, meeting with leaders, visiting friends and relatives, going out, attending religious services, attending groups/clubs, visiting others and socialising with co-workers and neighbours). Possible range from 9 to 45, with higher participation scores reflecting greater social participation.

g Social participation model adjusted for age, marital status and urban/rural residency.

h Urban/rural was adjusted for age.

#### Vegetable consumption

In both men and women, increasing age was associated with lower vegetable consumption (*β* = –0·06, 95 % CI = –0·07, –0·04 and *β* = –0·05, 95 % CI = –0·07, –0·03, respectively) (Table 3). When examined as a categorical variable compared with adults aged between 50 and 59 years, adults aged 60–69 years, 70–79 years and 80 years and older all had lower levels of vegetable consumption in both men and women (e.g. 80+ years: *β* = –2·22, 95 % CI = –2·72, –1·72 and 80+ years: *β* = –1·66, CI = –2·34, –0·99). Higher levels of education were associated with higher vegetable consumption after adjusting for age and urban or rural residency in both men and women (completed college/university/post-grad *v*. no formal education: *β* = 1·24, 95 % CI = 0·38, 2·10 and *β* = 1·24, 95 % CI = 0·57, 1·91). Wald tests found overall associations between categorical age and education for both men and women (age: F(1,48) = 62·64, *P* < 0·001 and (F(1,48) = 38·06, *P* < 0·001, respectively; education: F(1,48) = 9·05, *P* = 0·004 and F(1, 48) = 7·39, *P* = 0·009, respectively). For men, greater social participation scores after adjusting for age, marital status and rural residency (*β* = 0·06, 95 % CI = 0·01, 0·11) and rural residency for both men and women after adjusting for age (*β* = 1·63, 95 % CI = 1·14, 2·11 and *β* = 1·04, 95 % CI = 0·49, 1·59, respectively) were associated with higher vegetable consumption. After adjusting for age, being partnered was associated with increased vegetable consumption compared with being unpartnered. However, this association was only observed in women (*β* = 0·31, 95 % CI = 0·02, 0·59). No other significant associations were found in the regressions of individual and environmental characteristics and vegetable consumption for either men or women.

**Table 3.** Findings from linear regression analysis of association between socio-ecologic characteristics and vegetable consumption (serves/day) by sex in older adults from WHO SAGE China, 2007–2010 (weighted) (*n* 9541)

	Men (*n* 4450)		Women (*n* 5091)	
	Crude	Adjusted	Crude	Adjusted
	*β* (95 % CI)	*β* (95 % CI)	*β* (95 % CI)	*β* (95 % CI)
Age (continuous)	–0·06 (–0·07, –0·04), *P* < 0·001***	n/a	–0·05 (–0·07, –0·03), *P* < 0·001***	n/a
Age				
50–59 years	ref	n/a	ref	n/a
60–69 years	–0·43 (–0·78, –0·07), *P* = 0·020*		–0·42 (–0·69, –0·15), *P* = 0·003**	
70–79 years	–1·17 (–1·61, –0·74), *P* < 0·001***		–0·90 (–1·29, –0·51), *P* < 0·001***	
80+ years	–2·22 (–2·72, –1·72), *P* < 0·001***		–1·66 (–2·34, –0·99), *P* < 0·001***	
Education^a^				
No formal education	ref	ref	ref	ref
Less than primary	0·88 (0·26, 1·49), *P* = 0·006**	0·76 (0·09, 1·43), *P* = 0·026*	0·61 (0·07, 1·15), *P* = 0·027*	0·46 (–0·14, 1·06), *P* = 0·128
Completed primary	0·73 (0·35, 1·11), *P < 0·*001***	0·94 (0·47, 1·40), *P* < 0·001***	0·20 (–0·25, 0·66), *P* = 0·371	0·32 (–0·14, 0·78), *P* = 0·167
Completed secondary	0·88 (0·28, 1·49), *P* = 0·005**	1·27 (0·58, 1·96), *P* = 0·001**	0·39 (–0·10, 0·89), *P* = 0·118	0·58 (–0·04, 1·20), *P* = 0·065
Completed high school	0·59 (–0·13, 1·32), *P* = 0·105	1·33 (0·53, 2·12), *P* = 0·002**	0·41 (–0·29, 1·11), *P* = 0·246	0·82 (0·10,1·54), *P* = 0·026*
Completed college/university/post-grad	–0·19 (–0·99, 0·61), *P* = 0·640	1·24 (0·38, 2·10), *P* = 0·005**	0·54 (–0·065, 1·15), *P* = 0·079	1·24 (0·57, 1·91), *P* = 0·001**
Financial security^b^				
No	ref	ref	ref	ref
Yes	–0·32 (–0·67, 0·04), *P* = 0·081	–0·30 (–0·67, 0·07), *P = 0·*104	0·02 (–0·30, 0·35), *P* = 0·890	0·00 (–0·33, 0·34), *P* = 0·979
Home ownership^c^,^d^				
Family-owned	ref	ref	ref	ref
Rented	–1·12 (–2·04, –0·19), *P* = 0·019*	–0·07 (–0·83, 0·68), *P* = 0·846	–0·85 (–1·54, –0·17), *P* = 0·016*	–0·19 (–1·04, 0·67), *P* = 0·663
Other	–0·67 (–1·38, 0·04), *P* = 0·065	0·39 (–0·38, 1·16), *P = 0·*313	–0·53 (–0·93, –0·12), *P* = 0·012*	0·08 (–0·42, 0·58), *P* = 0·753
Marital status^e^				
Unpartnered	ref	ref	ref	ref
Partnered	0·39 (–0·09, 0·88), *P* = 0·110	0·10(–0·37, 0·57), *P* = 0·663	0·71 (0·45, 0·98), *P* < 0·001***	0·31 (0·02, 0·59), *P* = 0·036*
Social Participation^f^,^g^	0·11 (0·06, 0·16), *P* < 0·001***	0·06 (0·01, 0·11), *P* = 0·016*	0·07 (0·02, 0·13), *P* = 0·012*	0·04 (–0·01, 0·09), *P* = 0·122
Location^h^				
Urban	ref	ref	ref	ref
Rural	1·72 (1·23, 2·20), *P* < 0·001***	1·63 (1·14, 2·11), *P* < 0·001***	1·11 (0·57, 1·65), *P* < 0·001***	1·04 (0·49, 1·59), *P* < 0·001***

* *P* value < 0.05.

** *P* < 0.01.

*** *P* < 0.001.

a Education model adjusted for age and urban/rural residency.

b Financial security model adjusted for age, education, marital status and urban/rural residency.

c Home Ownership ‘Other’ refers to households where the home is not owned by family (or the respondent) and rent is not paid, for example in government-provided accommodation, living with friends or living with an unrelated carer.

d Home ownership model adjusted for age, education, financial security, marital status and urban/rural residency.

e Marital status model adjusted for age.

f The social participation score was calculated based on responses from nine categories (community meetings, meeting with leaders, visiting friends and relatives, going out, attending religious services, attending groups/clubs, visiting others and socialising with co-workers and neighbours). Possible range from 9 to 45, with higher participation scores reflecting greater social participation.

g Social participation model adjusted for age, marital status, and urban/rural residency.

h Urban/rural residency model adjusted for age.

## Discussion

The aim of this study was to identify the individual and environmental characteristics associated with fruit and vegetable consumption among Chinese men and women aged 50 years and over. In the current sample, being partnered, having a higher level of education, being financially secure, owning one’s home, residing in an urban location and having higher social participation scores were associated with higher mean fruit consumption for both men and women, while increasing age was associated with lower fruit consumption only among women. However, increasing age was associated with lower vegetable consumption for both men and women. In comparison, higher vegetable consumption was associated with a higher social participation score in men, being partnered in women, having a higher level of education and rural residency in both men and women. This exploratory study identifies groups of older adults who may be at-risk of low fruit and vegetable consumption in a country where few studies have investigated the determinants of fruit and vegetable intake.

Men and women had higher average consumption of vegetables than fruit in the current study. Similarly, a previous cross-sectional study conducted in Chinese adults aged 60 years and over reported a higher percentage met vegetable recommendations (34·8 %) compared with fruit recommendations (5·0 %)^(8)^. This is in contrast to studies conducted in HIC where more people usually consume more fruit than vegetables^(24,41)^. Although there has been a transition towards Chinese people adopting a more Westernised diet, previous research has suggested that older adults may be more resistant to change in dietary habits^(42,43)^. Therefore, the high vegetable consumption by the sample overall may represent a more traditional diet retained from earlier years.

Previous research suggests that men and women consume different amounts of fruit and vegetables; however, the majority of these were conducted in HIC^(14,17,19,21,22,25,30,31)^. The current study also found that men and women differed in their consumption of fruit and vegetables. Specifically, women were found to consume an additional half serve of fruit compared with men, whilst men consumed an additional half serve of vegetables compared with women. Both of these observations have been supported by previous research in Chinese older adults^(30)^.

An association between increasing age in adults and higher fruit consumption has previously been observed in middle-income countries^(26–30)^. The current study found increasing age was associated with lower fruit consumption but only among women. In addition, increasing age was associated with lower vegetable consumption in both men and women. Previous research has found similar results in high- and middle-income countries^(17,27,28)^. Increasing age is correlated with a decreased appetite and lower overall consumption of food, which may be contributing to the observed findings^(44)^. Poor oral health and edentulism are often observed with increasing age and is also a likely contributing factor^(45)^. This suggests there is variation in fruit and vegetable intake across the mid (50–64 yrs) and the multiple stages of older age from 65 to 79, 80 to 99 and 100+ years. Those in later stages of older age are likely to have different dietary behaviour risks and influences compared with those just entering older age and should not be treated as one homogenous group in efforts to support healthy dietary behaviours.

The current study also considered education levels, financial security and home ownership as markers of socio-economic status as characteristics potentially associated with fruit and vegetable intake. Both higher levels of education and being more financially secure were associated with higher fruit consumption in men and women, consistent with previous research^(13,15,16,22,24,24–32)^, including a previous study of older Chinese adults^(30)^. Interestingly, financial security was not associated with vegetable intake in the present study, although modest associations between vegetable intake and education in men and women were reported. This adds to previous research conducted in both HIC and middle-income countries that has demonstrated inconsistent associations between vegetable intake and income, which may suggest a socio-cultural variation^(17–19,26,30,31)^. This may be due to differences in the typical diets across countries by socio-economic groups, for example rural areas of China have traditionally consumed a diet rich in vegetables, much of it self-produced compared with other middle-income countries^(42)^.

This study found that older people who resided in a family-owned home reported higher fruit consumption compared with those who resided in a rented home. However, this was only true in the adjusted model. It is possible that marital status, financial security and education explain these; however, the mechanisms behind this are unclear. For example, people who own a home may be more likely to be financially secure and able to afford to purchase fruit. To date there has been limited investigation of home ownership as a determinant of fruit and vegetable consumption, with one UK study reporting that owning a home was associated with greater fruit and vegetable variety but not amount consumed, whilst no associations were discovered in two other UK studies between home ownership and fruit and vegetable intake^(13,15,16)^. Drawing conclusions from the current study and previous work should be done with caution as they were conducted in two separate countries (China and the UK, respectively), and there may be cultural differences in play. Furthermore, differences in dietary assessment (24-hour recall and FFQ) and definitions of home ownership may contribute to the heterogeneity between these studies, and more research is needed to understand home ownership as a dietary determinant. The present study found that older men and women who were partnered consumed more fruit than their un-partnered counterparts. Additionally, partnered women consumed more vegetables than un-partnered women. Previous studies have also shown that fruit consumption is higher among those who are married^(15,22)^. A previous longitudinal UK study investigating marital transitions in adults aged 39–78 years found that becoming widowed was associated with lower fruit and vegetable intake in men but not women^(46)^. Interestingly, the current study found marital status influenced fruit consumption across both men and women but vegetable intake only in women. The reasons for this are unclear, and more research is needed to understand the differences in influence of marital transitions on dietary behaviours in Chinese men and women.

Higher social participation was associated with higher fruit intake in men and women and vegetable intake in men, consistent with most previous research^(15,19,20,29)^. Previous research in the UK has produced mixed results, although a Taiwanese study found that fruit and vegetable intake (combined) was higher with increasing social support in men and women^(13,15,19,20,26)^. A previous longitudinal study conducted in the UK found that increased social participation and support was associated with better diet quality, however, did not have an effect on dietary change in older adults over a 10-year period^(47)^. It is unknown if there are any longitudinal effects of social participation on dietary quality or fruit and vegetable intake in China so further research is warranted.

Rural residency was associated with lower fruit but higher vegetable consumption in men and women in the current sample, consistent with a previous Chinese study in adults aged 60 years and over^(30)^. Rural Chinese residents have historically produced a large proportion of the food they consume and may be less likely to be affected by increasing commercialisation^(42)^. Traditionally, rural residents in China used agricultural land to produce vegetables and grain for consumption and fruits were not prioritised, which may explain this finding^(42)^. Although rural areas are becoming more commercialised, diversity of food products, especially fruits, is still limited^(48)^.

This study has several limitations. First, this was a cross-sectional study and so causal relationships could not be determined. Given the lack of data in low- and middle-income countries, these cross-sectional data are important as starting point for future research; however, longitudinal studies should be conducted in future research to investigate temporal relationships. Second, report of fruit and vegetable intake relied on memory and was self-reported, which may lead to recall bias and social desirability bias^(49,50)^. However, the SAGE survey has been validated in the current population and relies on relatively short-term recall. Third, only limited dietary behaviours were assessed in the SAGE survey. Therefore, characteristics associated with other dietary behaviours, which may be important for chronic disease or more comprehensive dietary pattern and diet quality measures could not be examined. As this was an exploratory analysis, no adjustment for multiple testing was undertaken so there is an increased chance of false-positive associations. However, coefficients, 95 % confidence intervals and *P* values to three decimal places are presented to allow readers to assess the strength of associations. Finally, participants with missing data were excluded from the analysis, which may have affected the representativeness of the sample. Overall, 3867 participants were excluded leaving 71·2 % of the sample for analysis. There were some differences in characteristics between included and excluded participants, for example, excluded respondents were older, had lower levels of education and larger proportion lived in rural locations (see online Supplementary Table 1).

This study also had a number of strengths. First, assessing fruit and vegetable consumption separately allowed the specific characteristics associated with each food group to be assessed, based on results from previous studies^(14–17,19–22,24,28)^. Additionally, survey weighting by age, sex and rural residency was included in the analysis increasing representativeness^(51)^. Moreover, the large sample size has allowed for an examination of men and women separately. Despite these strengths, the results should not be generalised to other countries as there may be specific socio-cultural and economic variations impacting lifestyle and dietary patterns. Future research should also investigate how individual and environmental characteristics associated with fruit and vegetable intake interact to develop a better understanding of the complex mechanisms impacting dietary behaviour.

### Conclusion

This study has indicated the sex-specific associations between a range of individual, social and physical environmental characteristics and fruit and vegetable consumption in a nationally representative sample of older Chinese men and women. Findings suggest that fruit consumption is associated with education level, financial security, marital status, social participation and rural residency in men and women and age in women, whereas vegetable consumption is associated with age, rural residency, social participation in men and marital status in women. This study provides insight into groups of the Chinese older adult population that are potentially at-risk of low fruit and vegetable consumption. The results may be useful in guiding future research and in the development of policy and interventions specifically targeted towards these potentially at-risk groups. Further research into the interrelationship of these characteristics, longitudinal effects and whole diet is required to better understand the influences on diet in low- and middle-income countries.

## Supporting information

Derbyshire et al. supplementary material Derbyshire et al. supplementary material

## Supplementary material

For supplementary material/s referred to in this article, please visit https://doi.org/10.1017/S000711452400151X
