# Supplementary material for: Characteristics associated with fruit and vegetable consumption in mid aged and older Chinese men and women: a cross-sectional analysis of the first wave of WHO SAGE China
Source: Br J Nutr. 2024 Oct 15;132(6):805–14. doi: 10.1017/S000711452400151X (PMC11557287; doi:10.1017/S000711452400151X)

**Supplementary Information**

Supplementary Table I. Descriptive characteristics of older adults in China, WHO SAGE Wave 1 2007-2010: Included and Excluded cases (n=13408)

|  | Included  (n=9541) | Excluded  (n=3867) | P-value |
| --- | --- | --- | --- |
| Age, y, mean (SD), range | 62.9 (9.3), 50-95 | 63.8 (9.9), 50-99  (n=3826) | <0.0001 |
| Gender, n(%) | | n=3828 |  |
| Men | 4450 (46.6) | 1824 (47.7) | 0.279 |
| Women | 5091 (53.4) | 2002 (52.3) |  |
| Fruit Intake, serves/day, mean (SD) | 2.4 (2.2) | 2.5 (2.2)  (n=2530) | 0.169 |
| Vegetable Intake, serves/day, mean (SD) | 6.9 (3.9) | 7.2 (4.2) | 0.0006 |
| Education, n (%) |  | n=3867 |  |
| No formal education | 2305 (24.2) | 1264 (32.7) | <0.001 |
| Less than primary | 1733 (18.2) | 616 (15.9) |  |
| Completed primary | 1883 (19.7) | 712 (18.4) |  |
| Completed secondary | 1998 (20.9) | 613 (15.8) |  |
| Completed high school | 1223 (12.8) | 461 (11.9) |  |
| Completed college/university/ post graduate | 399 (4.2) | 201 (5.2) |  |
| Financially Secure, n (%) |  | n=2920 |  |
| Yes | 6757 (70.8) | 2049 (70.2) | 0.50 |
| No | 2784 (29.2) | 871 (29.8) |  |
| Home Ownership, n (%) |  | n=3775 |  |
| Family-owned | 8439 (88.5) | 3561 (94.3) | <0.001 |
| Rented | 583 (6.1) | 94 (2.5) |  |
| Other | 519 (5.4) | 120 (3.2) |  |
| Marital Status, n (%) |  | n=3816 |  |
| Partnered | 7973 (83.6) | 3210 (81.8) | 0.012 |
| Un-partnered | 1568 (16.4) | 696 (18.2) |  |
| Social Participation | 14.9 (3.6) | 14.9 (3.6)  (n=3336) | 0.611 |
| Location, n (%) |  | n=3867 |  |
| Urban | 4808 (50.4) | 1777 (45.9) | <0.001 |
| Rural | 4733 (49.6) | 2090 (54.1) |  |
| Language |  | n=3435 |  |
| Chinese, Mandarin | 9508 (99.6) | 3423 (99.6) | 0.282 |
| Chinese, other | 27 (0.3) | 12 (0.4) |  |
| Other | 6 (0.1) | 0 (0) |  |
| Angina, n (%) |  | n=3411 |  |
| Yes | 1390 (14.6) | 523 (15.3) | 0.280 |
| No | 8151 (85.4) | 2888 (84.7) |  |
| Diabetes, n (%) |  | n=3381 |  |
| Yes | 623 (6.5) | 222 (6.6) | 0.941 |
| No | 8918 (93.5) | 3159 (93.4) |  |
| Stroke, n (%) |  | n=3407 |  |
| Yes | 468 (4.9) | 190 (5.6) | 0.125 |
| No | 9073 (95.1) | 3217 (94.4) |  |
| Disability Status, n (%) |  | n=2643 |  |
| Not | 7786 (81.6) | 2066 (78.2) | <0.001 |
| Severe/Extreme | 1755 (18.4) | 577 (21.8) |  |
| Self-Rated Health, n (%) |  | n=3414 |  |
| Good | 3210 (33.6) | 1163 (34.1) | 0.283 |
| Moderate | 4396 (46.1) | 1524 (44.6) |  |
| Bad | 1935 (20.3) | 727 (21.3) |  |
| BMI, kg/m^2^, mean (SD) | 23.7 (3.4) | 23.9 (3.4)  (n=2748) | 0.0377 |
| BMI, kg/m^2^, n (%) |  | n=2748 |  |
| Underweight | 423 (4.4) | 97 (3.5) | 0.073 |
| Normal | 3729 (39.1) | 1039 (37.8) |  |
| Increased risk | 4070 (42.7) | 1226 (44.6) |  |
| High risk | 1319 (13.8) | 386 (14.1) |  |
| Alcohol Consumption, n (%) |  | n=3368 |  |
| No, never | 6552 (68.7) | 2386 (70.8) | 0.021 |
| Yes, not recently | 987 (10.3) | 350 (10.4) |  |
| Yes | 2002 (21.0) | 632 (18.4) |  |
| Smoking Status, n (%) |  | n=3383 |  |
| No | 6942 (72.8) | 2496 (73.8) | 0.250 |
| Yes | 2599 (27.2) | 887 (26.2) |  |
| Physical Activity (moderate-vigorous), min/week, mean (SD) | 706 (718) | 561 (714)  (n=3094) | <0.0001 |
| Sedentary Behaviour, min/week, mean (SD) | 1558 (939) | 1634 (957)  n=3094 | 0.0001 |

**Supplementary Figures 1-7:** Directed Acyclic Graph Adjustment Models for exposures age, education, financial security, home ownership, marital status, social participation, and urban/rural location

**Figure 1**


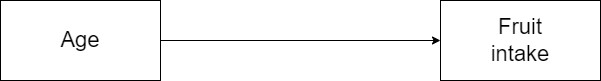


**Figure 2**


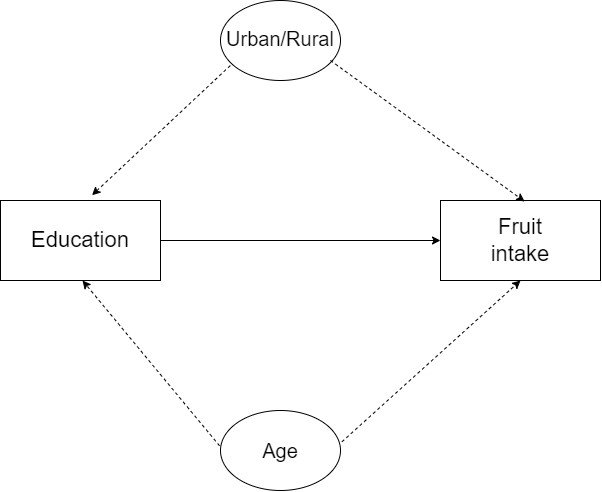


**Figure 3**


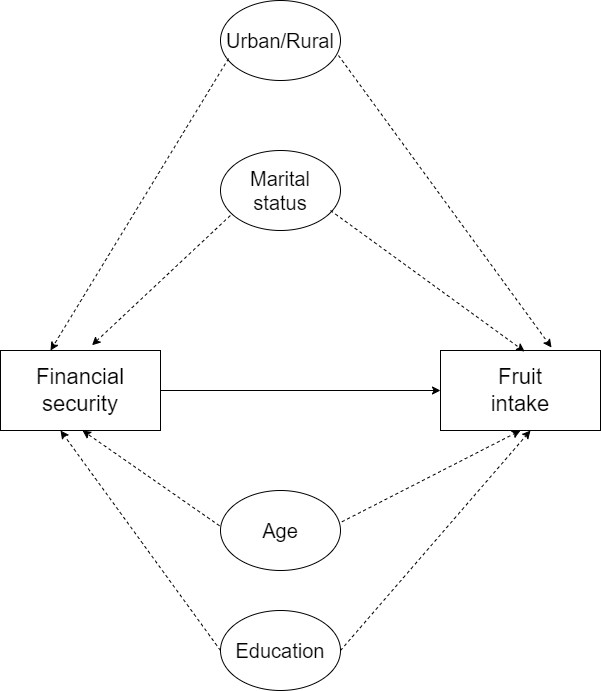


**Figure 4**


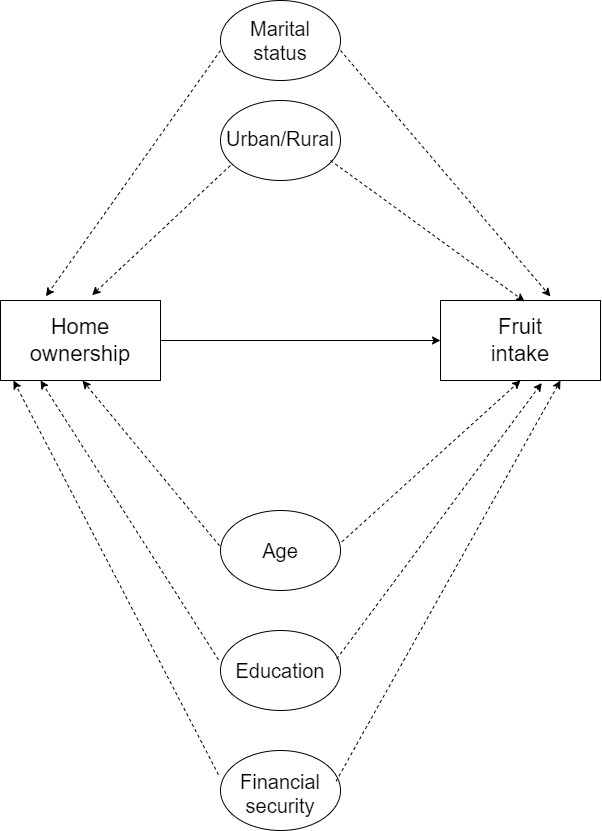


**Figure 5**


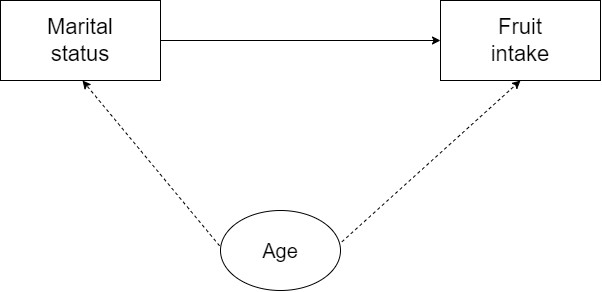


**Figure 6**


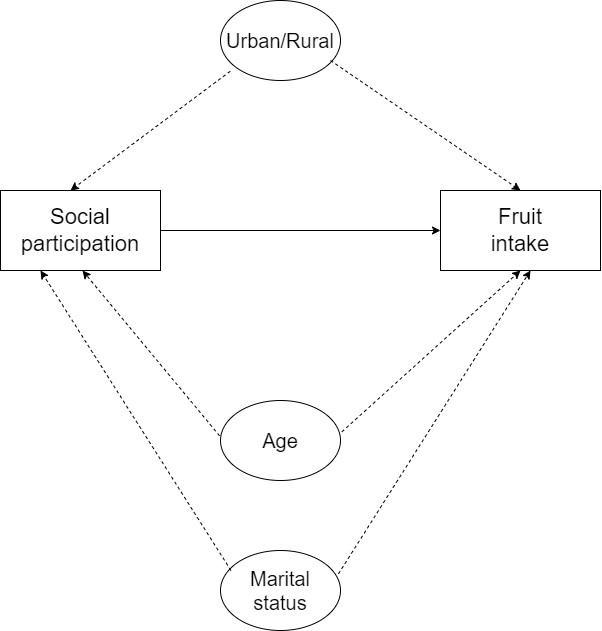


**Figure 7**


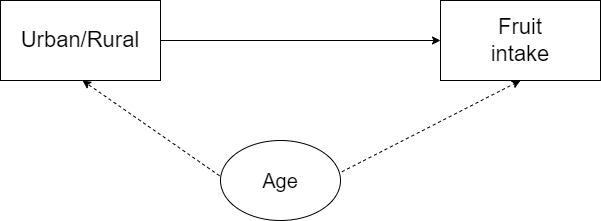

Supplement: Derbyshire et al. supplementary material [file S000711452400151Xsup001.docx]
